# Two Cases of Protruding Thrombus in the Ascending Aorta

**DOI:** 10.3400/avd.cr.20-00155

**Published:** 2021-03-25

**Authors:** Noriyuki Abe, Ken Yasumori, Noriko Shimabukuro, Takahiro Yamazato, Hiroshi Munakata

**Affiliations:** 1 Department of Cardiovascular Surgery, Okinawa Nanbu Prefectural Medical Center and Children’s Medical Center, Naha, Okinawa, Japan

**Keywords:** thrombus, ascending aorta, platelet

## Abstract

In the first case, a 60-year-old man was referred to our hospital for a sudden stomachache. A computed tomography scan revealed a thrombus at ascending aorta with acute mesenteric ischemia. In the second case, a 62-year old man developed a hypoglycemic attack with unbalanced diet. A computed tomography showed a thrombus at ascending aorta without thromboembolism. Laboratory data of both cases showed elevated platelet and a loss of antithrombin III. We administered a resection of thrombus to prevent a systemic embolism. We suggested that the risk of ascending aorta thrombus was elevated platelet and a loss of antithrombin III.

## Introduction

A localized thrombus involving the ascending aorta rarely occurs in the absence of an underlying cause, such as chest trauma, atherosclerosis, a hypercoagulable state, or instrumentation.^1)^ Conversely, the cause of thrombus was not often revealed. A high elevated platelet was suggested risk of an arterial thrombus.^2)^ We reported the two cases of a protruding thrombus at the ascending aorta.

## Case.1

**Figure 1:**
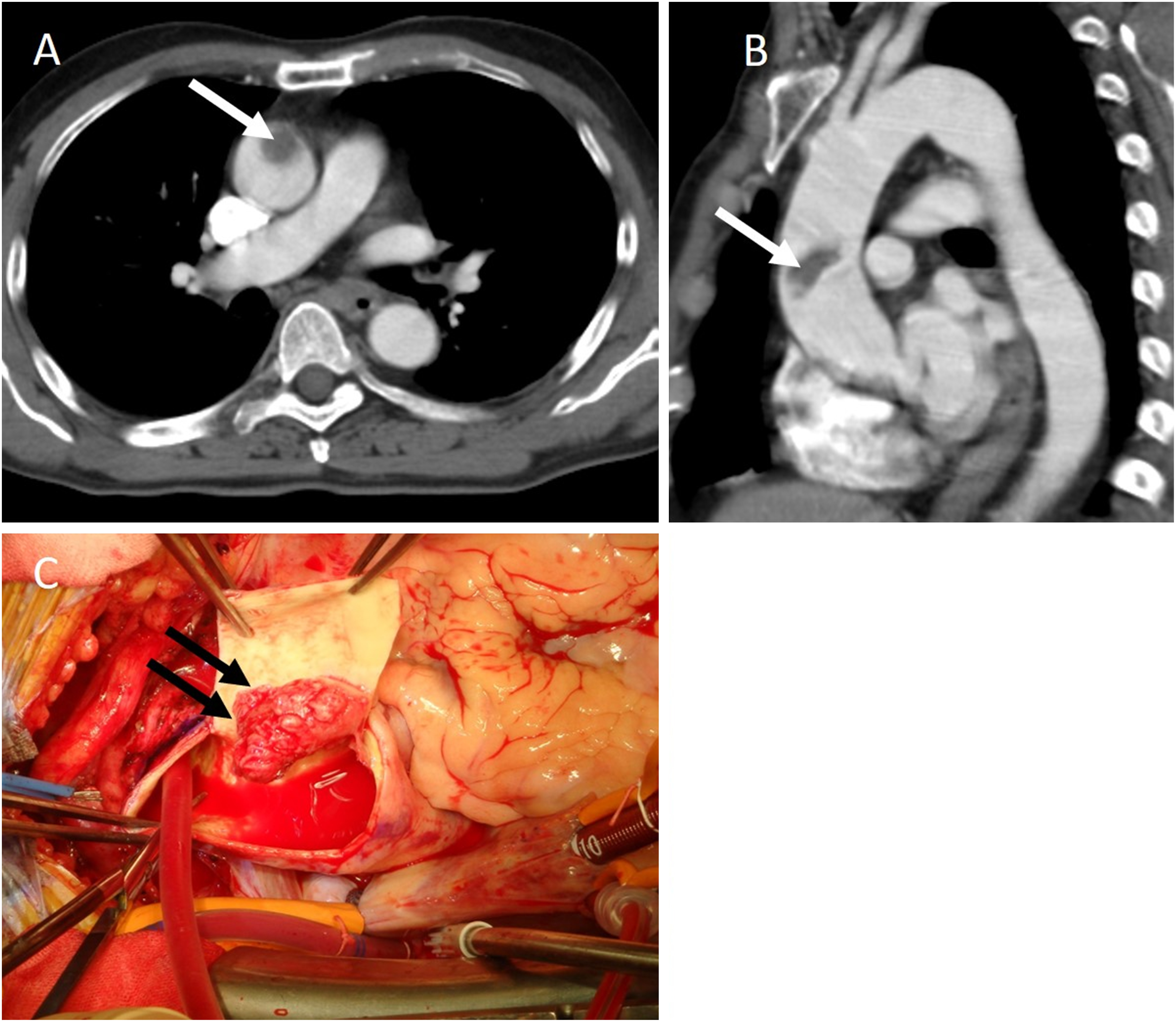
Fig. 1 (**A**, **B**) A contrast computed tomography showed thrombosis in ascending aorta (arrows). (**C**) In operative findings, the thrombus was size of 25×40 mm, fragile and high mobile (double arrows).

A 60-year-old man was referred to our hospital for a sudden stomachache. He presented with no previous history of thrombosis; he received an endoscopic mucosal resection for colon cancer two weeks prior. The pathological diagnosis was tubular adenocarcinoma, a well-differentiated type with submucosal layer and no vascular invasion, with a negative specimen stump. A computed tomography scan simultaneously revealed a protruding tumor at ascending aorta and an embolism in the superior mesenteric artery (**Figs. 1A** and **1B**). An electrocardiogram showed sinus rhythm. Laboratory data showed elevated platelet, a loss of antithrombin III (63%), and mild liver dysfunction. No tumor was present in the heart, and the cardiac function was normal based on the echocardiography results. He was diagnosed was acute superior mesenteric artery occlusion and ascending aortic thrombus.

**Figure 2:**
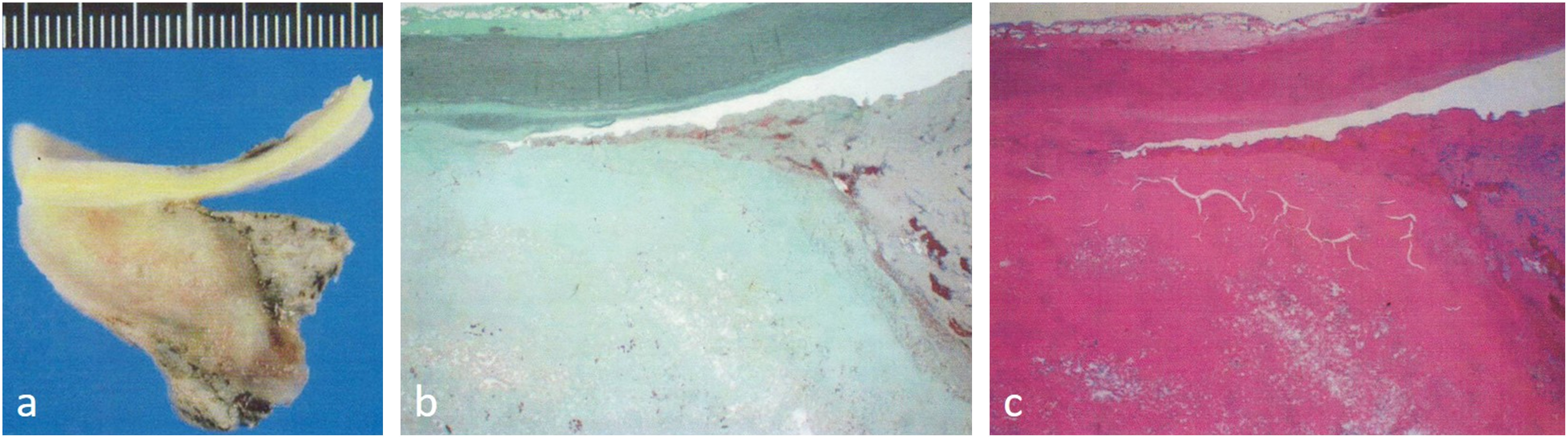
Fig. 2 The pathological findings showed thrombus originating from intimal laminae. The joint surface with aortic wall and thrombus was clear, and the ascending aorta showed no plaque and no malignancy.

We surgically resected the thrombus at ascending aorta after bowel resection. Intraoperative transesophageal echocardiography revealed the protruding thrombus (22.5×15.7 mm in size) to be mobile with a smooth surface. With supported cardiopulmonary bypass, the tumor and ascending aorta was resected under circulation arrest and selective antegrade cerebral perfusion. The thrombus was size of 25×40 mm, fragile and high mobile (**Fig. 1C**). The ascending aortic wall was a smooth surface and less arteriosclerotic change. A replacement of the ascending aorta was performed using a prosthesis graft [J Graft SHIELD NEO, 26 mm (Japan Lifeline Co., Ltd., Tokyo, Japan)]. Postoperative laboratory data showed a decline of antithrombin III (67%) and normal platelet. The patient survived for 2 years postoperatively. The pathological findings indicated a thrombus originating from intimal laminae. The joint surface of the aortic wall and thrombus was clear, and the ascending aorta showed no plaque or malignancy (**Fig. 2**). Postoperatively, warfarin was administered.

## Case.2

**Figure 3:**
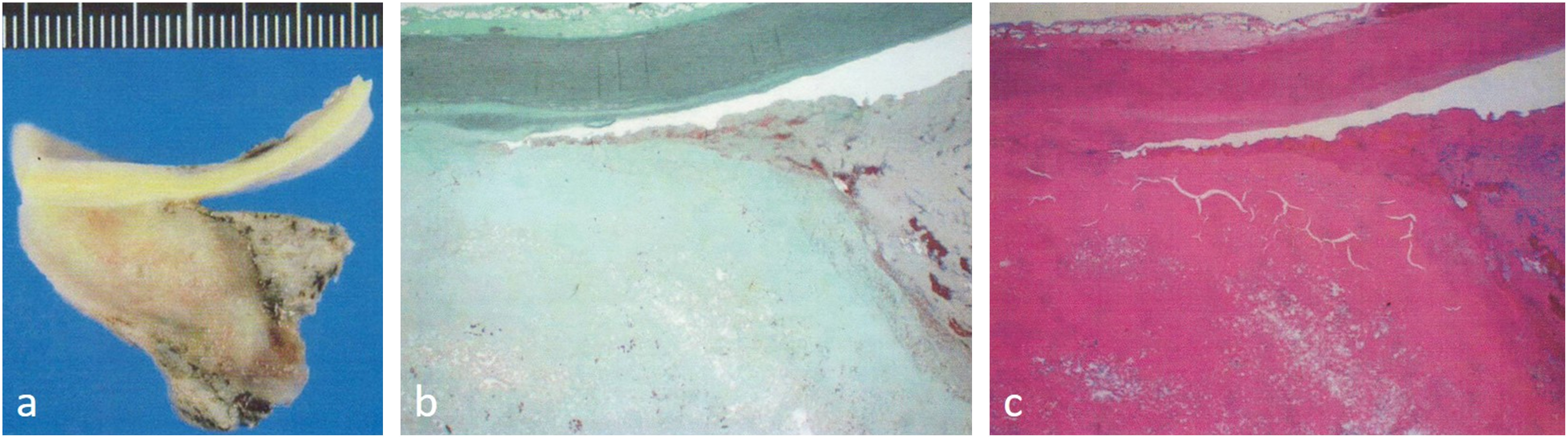
Fig. 3 (**A**) A contrast computed tomography showed thrombus in ascending aorta (arrow). (**B**) In operative findings, protruding thrombosis (16×10 mm: double arrows) in ascending aorta.

A 62-year old man was referred to our hospital for a hypoglycemic attack with unbalanced diet. Laboratory data showed high elevated platelet and a loss of antithrombin III (72%). In additional examination, a computed tomography showed a thrombus as a 10×11 mm at ascending aorta without thromboembolism (**Fig. 3A**). We decided to perform a resection of thrombus to prevent a systemic embolism. Under the cardiopulmonary bypass, the ascending aorta was clamped proximal to the brachiocephalic artery because the thrombus was confirmed by an intraoperative direct echocardiography (movie). The thrombus with ascending aorta was resected. The thrombus exhibited a size of 10×11 mm, high mobile and fragile (**Fig. 3B**). The aortic wall was smooth surface and less arteriosclerotic change. A replacement of the ascending aorta was performed using a prosthesis graft [Triplex, 24 mm (Terumo Co., Tokyo, Japan)]. Postoperative laboratory data showed a decline of antithrombin III (70%) and normal platelet. The patient was in good condition for 12 months postoperatively. The pathological findings showed a thrombus without malignancy. Warfarin was administered for 3 months after the operation.

## Discussion

In both cases, the differential diagnosis was arrested thrombus, vegetation, malignant tumor, etc. The vegetation was excluded because of no infectious symptoms. The characteristics of the malignant tumor were a wide basis and hard adhesions.

A previous study reported that the cause of an ascending aorta thrombus could be chest trauma, atherosclerosis, a hypercoagulable state, or instrumentation.^1)^ The etiology of a thrombus in the aorta is complex. Aortic thrombi can be caused by blood disorders (e.g., protein S or protein C deficiency and anti-phospholipid antibody syndrome), tumors, aortitis, collagen disease, aortic structural abnormalities (e.g., aortic aneurysms), intra-aortic atheroma, hormone therapy, steroid use, and atrial fibrillation.^3)^ In both cases, electrocardiogram showed sinus rhythm. No previous trauma or a supported device was found. Laparche et al. reported that risk factors of mobile thrombus in the aortic arch included heavy smoking, hypercholesterolemia, and elevated fibrinogen (> 400 mg/dl).^4)^ In addition, the hypercoagulable state related with malignant tumor has been referred to as Trousseau’s syndrome, one of the paraneoplastic syndromes.^5,6)^ One case suggested that it might be related with a malignant tumor. In both cases, laboratory data showed high elevated platelet and a loss of antithrombin III (Supplemental). Because disease was not obvious, blood agglutination by dehydration can cause thrombus.^2)^

Because a surgical indication of ascending aorta thrombus was risk of embolism, these cases were performed in emergency operation. The previous study reported that endovascular therapy may be a useful option for descending thoracic aortic thrombus.^7)^ In addition, few case reports of endovascular treatment for ascending aortic thrombus exist.^8)^

Our strategy of procedure was resection of all thrombus and aortic wall all together. In the previous report, a floating thrombus in the ascending aorta was suggested to be associated with atherosclerosis of the aortic wall. We replaced ascending aorta with prosthesis graft in order to prevent recurrence and systemic embolism.^3,9)^ One case was clamp and another case was non-clamp. The difference in these cases was the site of thrombus. If the thrombus was near brachiocephalic artery, a non-clamp procedure was selected. We were able to avoid recurrent embolism. The pathological findings revealed that the thrombus originating from intimal laminae and the joint surface was clear, and the ascending aorta showed no plaque or malignancy.

## Conclusion

We reported an extremely rare two cases where surgical repair was performed for an ascending aorta thrombus. We proved that the thrombus originated in the intimal laminae with edge clarity by pathological diagnosis. Lastly, we suggested that the risk of ascending aorta thrombus was elevated platelet and a loss of antithrombin III.

## Additional Note

Because this study followed the ethical standards of the institution, this work does not require ethical considerations.

## Supplementary Information

Supplementary movie is available at the online article site on J-STAGE and PMC.
